# 5-aza-2′-Deoxycytidine Induces a RIG-I-Related Innate Immune Response by Modulating Mitochondria Stress in Neuroblastoma

**DOI:** 10.3390/cells9091920

**Published:** 2020-08-19

**Authors:** Hung-Yu Lin, Jiin-Haur Chuang, Pei-Wen Wang, Tsu-Kung Lin, Min-Tsui Wu, Wen-Ming Hsu, Hui-Ching Chuang

**Affiliations:** 1 Department of Internal Medicine, Kaohsiung Chang Gung Memorial Hospital and Chang Gung University College of Medicine, Kaohsiung 833, Taiwan; linhungyu700218@gmail.com (H.-Y.L.); wangpw@cgmh.org.tw (P.-W.W.); 2 Center for Mitochondrial Research and Medicine, Kaohsiung Chang Gung Memorial Hospital and Chang Gung University College of Medicine, Kaohsiung 833, Taiwan; jhchuang@adm.cgmh.org.tw (J.-H.C.); tklin@adm.cgmh.org.tw (T.-K.L.); cat2lily@gmail.com (M.-T.W.); 3 Research Assistant Center, Show Chwan Memorial Hospital, Changhua 500, Taiwan; 4 Department of Pediatric Surgery, Kaohsiung Chang Gung Memorial Hospital and Chang Gung University College of Medicine, Kaohsiung 833, Taiwan; 5 Department of Neurology, Kaohsiung Chang Gung Memorial Hospital and Chang Gung University College of Medicine, Kaohsiung 833, Taiwan; 6 Department of Surgery, National Taiwan University Hospital, Taipei 100, Taiwan; billwmhsu@gmail.com; 7 Department of Otolaryngology, Kaohsiung Chang Gung Memorial Hospital and Chang Gung University College of Medicine, Kaohsiung 833, Taiwan

**Keywords:** neuroblastoma, DNA methyltransferase inhibitor, innate immune response, mitochondria, double-stranded RNA

## Abstract

Background: Neuroblastoma (NB) is one of the most common malignant solid tumors to occur in children, characterized by a wide range of genetic and epigenetic aberrations. We studied whether modifications of the latter with a 5-aza-2′-deoxycytidine (decitabine, Dac) DNA methyltransferase inhibitor can provide a therapeutic advantage in NB. Methods: NB cells with or without *MYCN* amplification were treated with Dac. We used flow cytometry to measure cell apoptosis and death and mitochondrial reactive oxygen species (mtROS), microarray to analyze gene expression profile and bisulfite pyrosequencing to determine the methylation level of the *DDX58*/RIG-I promoter. Western blot was used to detect markers related to innate immune response and apoptotic signaling, while immunofluorescent imaging was used to determine dsRNA. We generated mtDNA depleted ρ^0^ cells using long-term exposure to low-dose ethidium bromide. Results: Dac preferentially induced a RIG-I-predominant innate immune response and cell apoptosis in SK-N-AS NB cells, significantly reduced the methylation level of the *DDX58*/RIG-I promoter and increased dsRNA accumulation in the cytosol. Dac down regulated mitochondrial genes related to redox homeostasis, but augmented mtROS production. ρ^0^ cells demonstrated a blunted response in innate immune response and apoptotic cell death, as well as greatly diminished dsRNA. The response of NB cells to CDDP and poly(I:C) was potentiated by Dac in association with increased mtROS, which was blunted in ρ^0^ cells. Conclusions: This study indicates that Dac effectively induces a RIG-I-related innate immune response and apoptotic signaling primarily in SK-N-AS NB cells by hypomethylating *DDX58*/RIG-I promoter, elevated mtROS and increased dsRNA. Dac can potentiate the cytotoxic effects of CDDP and poly(I:C) in NB cells.

## 1. Introduction

Neuroblastoma (NB) is the most common extracranial solid tumor to occur in children and is responsible for about 15% of pediatric oncology deaths [1]. Risk factors include age older than 18 months at diagnosis, advanced stage, unfavorable histologic grade and *MYCN* amplification. *MYCN*-amplified NB is highly correlated with advanced disease stage and poor prognosis, which accounts for 20–25% of overall and 40% of high-risk cases [2] and *MYCN*-nonamplified NB with elevated *c-MYC* expression is also associated with a poor prognosis in NB [3].

In addition to genetic abnormalities, epigenetic aberrations play an important role in the progression of NB. Epigenetic changes that occur in both single genes and at the genome-wide level. Hypermethylation in the promoter region of tumor suppressor genes is associated with poor outcome [4,5,6,7]. Genome-wide analysis of DNA methylation has revealed a DNA methylator phenotype in NB with poor prognosis, characterized by the methylation of a subset of multiple CpG islands [8,9]. Tumorigenic properties of NB can be inhibited by reversing epigenetic changes with DNA methyltransferase inhibitor 5-aza-2′-deoxycytidine (decitabine, Dac) [10], which is also FDA-approved for treating hematological malignancies [11]. Treatment of NB cells with Dac induced cell differentiation and reduced proliferation and colony formation [12,13]. Further studies demonstrated that Dac can potentiate the cytotoxic effects of current chemotherapies [14]. However, the molecular mechanism underlying the clinical effects of Dac remains uncertain. The reactivation of aberrantly methylated tumor suppressor genes following promoter demethylation has shown to grant an antitumor effect [15]. More recently, however, a couple of studies have demonstrated that the tumor-suppressing effect of Dac can be attributed to an activated innate immune response, in which an increase of endogenous dsRNA stimulates retinoic acid-inducible gene I (RIG-I) and melanoma differentiation-associated protein 5 (MDA5) and can then trigger mitochondrial antiviral signaling protein (MAVS)/interferon regulatory factor 3 (IRF3) pathway, ultimately leading to cell death [16,17].

Mitochondria are responsible for the cellular bioenergetics and are involved in redox status. Mitochondrial DNA (mtDNA) encodes tRNA, rRNA and proteins that are essential for oxidative phosphorylation (OXPHOS). This versatile organelle, which includes mtDNA and other interior components and associated proteins, constitutes a central hub of innate immune signaling [18]. The integrity of mitochondrial DNA (mtDNA) plays a central role in MAVS-related pathway activity in HeLa cells [19,20]. In fact, our previous study demonstrated that mtDNA is involved in TLR3-agonist induced oxidative stress and cell death in NB [21]. In this study, we demonstrated that Dac induces a RIG-I-associated innate immune response and cell death in NB through hypomethylated *DDX58*/RIG-I promoter and accumulated endogenous dsRNA. We also verified the involvement of mitochondria in mediating the anti-NB effect of Dac by using the ρ^0^ cell devoid of mtDNA. Finally, we found that Dac can potentiate the anti-NB effect of cisplatin and/or poly(I:C), which are known for targeting mitochondria and stimulating innate immunity, respectively.

## 2. Materials and Methods

### 2.1. Cell Culture

Human NB cell lines (SK-N-AS, SK-N-FI, BE(2)-M17 and SKN-DZ) were purchased from the American Type Culture Collection (Manassas, VA, USA). All cell lines were maintained in Dulbecco’s modified Eagle’s medium (DMEM) (12100-046, Thermo Fisher Scientific, Waltham, MA, USA) and supplemented with 10%-heat-inactivated fetal bovine serum (FBS; 10437-028, Thermo Fisher Scientific, Waltham, MA, USA), GlutaMAX (35050-061, Thermo Fisher Scientific, Waltham, MA, USA), non-essential amino acids (11140-050, Thermo Fisher Scientific, Waltham, MA, USA) and an antibiotic–antimycotic (15240-062, Thermo Fisher Scientific, Waltham, MA, USA) in a 5%-CO_2_ humidified incubator at 37 °C. NB cells treated with 5-aza-2′-deoxycytidine (Dac) (A3656, Sigma-Aldrich, St. Louis, MO, USA) at various doses were harvested after 3 or 5 days. For synergistic treatment with other drugs, NB was first grown in medium containing 2.5-μM Dac for 3 or 5 days. Then, Dac-containing medium was washed out and replaced with fresh medium loaded with 50-μg/mL poly(I:C)(tlrl-pic-5, Invivogen, San Diego, CA, USA), 10-μM cisplatin (Fresenius Kabi India, Pune, India) or a combination of both.

### 2.2. Gene Expression Microarray Assay

Collected RNA samples were subjected to microarray assay to determine a gene expression profile. We utilized Affymetrix Clariom D microarray chips for profiling. The RNA sample were first prepared using the WT PLUS reagent kit (Affymetrix, Thermo Fisher Scientific, Waltham, MA, USA) followed by hybridization on the Clariom D microarray chips. The raw data of Clariom D chips were first subjected to quality control examination pursuant to the Affymetrix manuals. The chips that passed the quality control criteria were then analyzed with Partek; a commercial software specific for microarray data analysis.

### 2.3. Methylation Analysis Using Bisulfite Pyrosequencing

Pyrosequencing was conducted for four CpGs sites within the RIG-I/*DDX58* promoter region. Briefly, 500 ng of each genomic DNA sample was bisulfite-converted using the EpiTect Plus DNA bisulfite kit (Qiagen, Hilden, Germany). The primer sequences used for bisulfate pyrosequencing are listed in Supplementary Table S1. The PCR program was 95 °C for 5 min, 40 cycles of 94 °C for 30 s, 56 °C for 30 s and 72 °C for 30 s, followed by a final extension at 72 °C for 10 min. Single-stranded DNA templates were prepared from the biotinylated PCR product using streptavidin-coated sepharose beads (streptavidin sepharose high performance, GE Healthcare, Inc., Chicago, IL, USA), where the sequence primer was annealed. Primed templates were sequenced using the PyroMark Q24 System (Qiagen, Inc.) and the assay setup was generated using PyroMark Q24 Application Software 2.0 (Qiagen, Inc.).

### 2.4. Gene Knockdown

Knockdown of *DDX58*/RIG-I was conducted by using small-interfering RNA (siRNA). Control scramble siRNA (si-CON) and si-RIG-I were purchased from (D-001810-10-20 and L-012511-00-0020, respectively, Horizon Discovery, Cambridge, UK). Gene delivery of siRNA into cells was conducted using lipofectamine RNAiMAX (Invitrogen, Carlsbad, CA, USA) in accordance with the manufacturer’s protocol. Briefly, 5 × 10^6^ cells were seeded in 100-mm culture dishes 24 h before transfection. Then, 25-nM siRNA in 7.5 μL DEPC water (tube 1) and 20 μL of lipofectamine RNAiMAX reagent (tube 2) were separately diluted in 500 μL serum-free Opti-MEM (Invitrogen, Carlsbad, CA, USA), followed by 15–20 min incubation at room temperature. Cultured cell then underwent 6 h of incubation with siRNA-mix, followed by 18 h of incubation with growth medium. Finally, cells were divided into no-treated and Dac-treated groups for subsequent 5 days.

### 2.5. MtDNA-Devoid ρ^0^ Cells

The procedure for generating mtDNA-devoid SK-N-AS cells (ρ^0^) has previously been described. Briefly, cells were treated with 50-ng/mL ethidium bromide for 12 weeks in the presence of 1-mM pyruvate and 50-μg/mL uridine. Limit dilution was employed to obtain single and stable ρ^0^ clones. The mtDNA-depletion status was characterized by mtDNA copy number, mtDNA-coded protein (cytochrome c oxygenase subunit 2, COX2) expression and inviable phenotype under a medium free of pyruvate and uridine.

### 2.6. MtDNA Copy Number

We determined mtDNA content using real-time PCR (Light-cycler 480, Roche, Basel, Switzerland). To determine content of nuclear DNA as a copy number reference, we used the forward primer 5′-GGCTCTGTGAGGGATATAAAGACA-3′ and reverse primer 5′-CAAACCACCCGAGCAACTAATCT-3′, both of which were complementary to the sequences of the chromosome 1 genome loci on 1q24-25. To analyze the mtDNA content, we used the forward primer 5′-CACAGAAGCTGCCATCAAGTA-3′ and reverse primer 5′-CCGGAGAGTATATTGTTGAAGAG-3′, both of which were complementary to the sequences of ND2. The difference of threshold cycle number (∆Ct) values of the nuclear chromosome 1 gene and the mitochondrial ND2 gene were calculated during each PCR run. The mitochondrial copy number was calculated using the following formula:

Copy number (copies/cell) = 2 × 2^∆Ct^ (1)

### 2.7. Western Blotting

Proteins from no-treated control and drug-treated samples were separated in 8–12% SDS-PAGE gels and were transferred onto 0.45-μm PVDF membranes (Millipore, Burlington, MA, USA) in a Trans-Blot^®^ SD Semi-Dry Transfer Cell (Bio-Rad) for 50 min at 400 mA. The membrane was blocked in 5% non-fat milk powder/PBS-T (1X PBS, 0.1% Tween-20 (Sigma-Aldrich, St. Louis, MO, USA) and incubated overnight at 4 °C with a blocking buffer containing primary antibodies, including anti-MDA5 (5321S, Cell Signaling, Danvers, MA, USA), anti-RIG-I (3743S, Cell Signaling, Danvers, MA, USA), anti-TLR3 (ab62566, Abcam, Cambridge, UK), anti-p-IRF3 (ab76493, Abcam, Cambridge, UK), anti-MAVS (sc-166583, Santa Cruz Biotechnology, Dallas, TX, USA), anti-c-PARP (9532S, Cell Signaling, Danvers, MA, USA), anti-c-caspase3 (9661S, Cell Signaling, Danvers, MA, USA), anti-c-caspase9 (7237S, Cell Signaling, Danvers, MA, USA), anti-c-caspase8 (9496S, Cell Signaling, Danvers, MA, USA) and anti-Tom20 (sc-17764, Santa Cruz Biotechnology, Dallas, TX, USA). The membrane was washed and then incubated for 1 h with 5% non-fat milk powder/PBS-T containing anti-rabbit IgG antibodies or anti-mouse IgG antibodies and was then washed and imaged with enhanced chemiluminescence (PerkinElmer, Waltham, MA, USA). The membrane images were analyzed using an AutoChemi image system (UVP) or exposed to Fuji medical X-ray film, followed by quantification with Alpha View SA 3.4.0 (ProteinSimple, San Jose, CA, USA).

### 2.8. Flow Cytometry Detecting Cell Death and ROS

The percentage of cell death was determined using propidium iodide (PI) (Sigma-Aldrich, St. Louis, MO, USA) and trypan blue (TB) staining, followed by cytometry-based analysis on the FL2 and FL3 channel, respectively. Briefly, cells were suspended in PBS and stained with PI or TB for 15 min at room temperature. The mitochondrial ROS was measured by MitoSOX^TM^ Red (Invitrogen, Carlsbad, CA, USA). Cells were washed twice with PBS and stained with MitoSOX™ red (5 μM) for 30 min at 37 °C. Then cells were collected, washed twice with PBS and finally resuspended in a flow tube with 1 mL PBS. The fluorescent signal of cell suspension was then measured using a FACS caliber 101 flow cytometer (BD Biosciences, San Jose, CA, USA) and analyzed using winMDI software.

### 2.9. Immunofluorescent Imaging

We seeded 1 × 10^4^ cells on an 18-mm cover glass (GMA81-018, MATSUNAMI, Bellingham, WA, USA). After being treated with 2.5-μM Dac for 5 days, cells were fixed in 4% formaldehyde, washed and then permeabilized with 0.25% Triton X-100 in PBS for 10 minutes. Cells were subjected to staining with primary antibodies mouse anti-dsRNA mAb J2 (1:200, 10010200; SCICONS, Szirák, Hungary) for 2 h and rabbit anti-Tom20 pAb (1:100; sc11415, Santa Cruz Biotechnology, Dallas, TX, USA) for 1 h, secondary antibodies Alexa 488-conjugated donkey anti-mouse (1:500, ab96873, Abcam, Cambridge, UK) and Alexa 594-conjugated goat anti-rabbit (1:500, ab96883, Abcam, Cambridge, UK) for 1 h and DAPI (D1306, Thermo Fisher Scientific, Waltham, MA, USA). After washing twice with PBS, cover glass was mounted with Fluoromount-G^®^ (0100-01, SouthernBiotech, Birmingham, AL, USA). Super-resolution imaging was performed using a confocal microscope (LSM 980 with Airyscan 2, Zeiss, Oberkochen, Germany). The fluorescence signal intensity of dsRNA was captured under lower magnification (100×) of the Olympus FV10i confocal microscope. Quantification of the dsRNA signal was obtained in three fields containing at least 17 cells for each group from three independent experiments. The quantification of fluorescent signal representing dsRNA level was normalized to cell number.

### 2.10. Statistical Analysis

Data expressed as the mean ± SEM were collected from at least three independent experiments. Differences between two data sets were evaluated using two-tailed unpaired Student’s *t*-test. Statistical tests between multiple data sets were analyzed using a one-way analysis of variance (ANOVA) followed by post hoc Tukey’s test. A *p*-value < 0.05 was considered statistically significant.

## 3. Results

### 3.1. Dac Preferentially Induces a RIG-I-Related Innate Immune Response and Cell Apoptosis in MYCN Non-Amplified SK-N-AS NB

**Figure 1:**
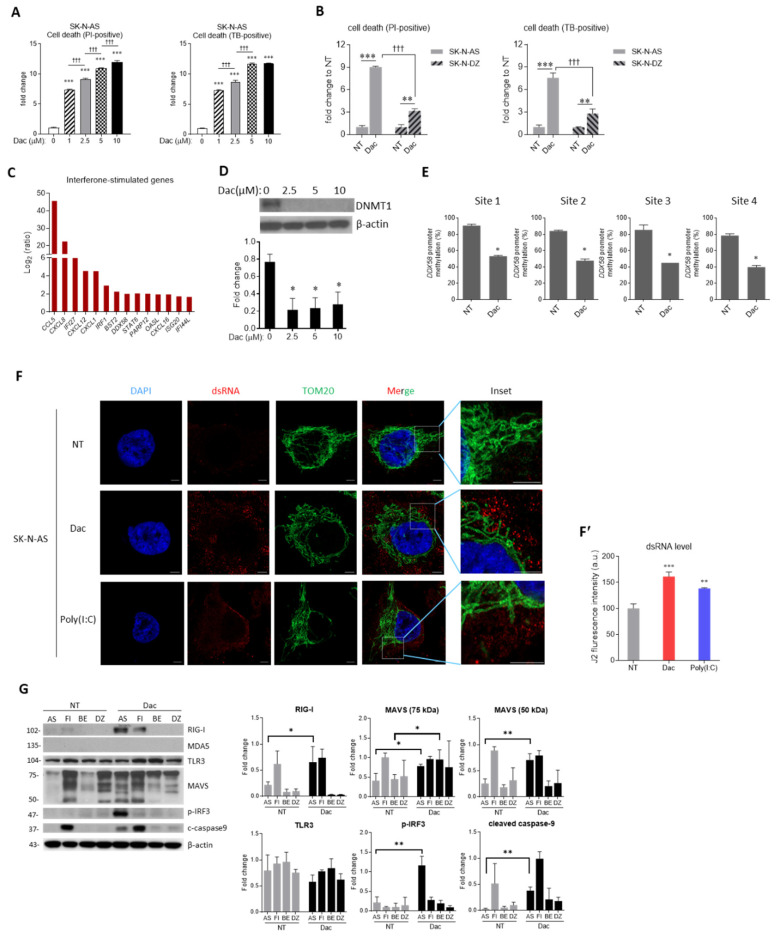
5-aza-2′-deoxycytidine (Dac) preferentially induces a retinoic acid-inducible gene I (RIG-I)-predominant innate immune response and cell apoptosis Human NB cells (SK-N-AS). (**A**) SK-N-AS cells were treated with Dac from 0 to 10 μM for 5 days. Cell death was determined using propidium iodide (PI) and trypan blue (TB) staining with the flow cytometry. ^†^ *p* < 0.05, ^†††^ *p* < 0.001 between indicated groups; (**B**) cell death of human NB cell lines SK-N-AS and SK-N-DZ cells treated with 2.5 μM Dac for 5 days; (**C**) SK-N-AS cells treated with 2.5 μM Dac or untreated control (NT) for 5 days were harvested and subjected to microarray analysis. Histogram showing up-regulated interferon-stimulated genes; (**D**) representative western blot of DNA methyltransferase 1 (DNMT1) and densitometric data; (**E**) SK-N-AS cells were treated with or without 2.5-μM Dac or NT for 5 days. Methylation level of four CpG sites at the *DDX58*/RIG-I promoter; (**F**) SK-N-AS cells were treated with 2.5-μM Dac or NT for 5 days. Representative immunofluorescent image of dsRNA (red), mitochondrial outer membrane marker TOM20 (green) and DAPI-stained nuclei (blue). Poly(I:C), a synthetic analog of dsRNA, was used as positive control. Scale bar, 5 μm. Insets contain magnified images highlighting mitochondria and dsRNA. Note that Dac treatment causes an increased level of dsRNA in cytoplasm. (**F’**) percentage of J2 fluorescence intensity (a.u., arbitrary unit) was quantified (representative images shown in Supplementary Figure S3B) under relatively lower magnification (100X) of an Olympus FV10i confocal microscope; (**G**) representative western blot result of proteins related to innate immune response and apoptotic signaling in various NB cells with (BE and DZ) or without (AS and FI) *MYCN* amplification. β-actin serves as loading control. Data shown as mean ± SD. * *p* < 0.05, ** *p* < 0.01 *** *p* < 0.001 when compared to 0-μM or NT group. NT—untreated.

In the study by Ikegaki et al. [22], they demonstrated that the epigenetic modifier Dac could induce the stemness phenotype of NB cells under five days of treatment. Therefore, we determined the stemness or cytotoxic effect of Dac 2.5-μM for 5 days on SK-N-AS NB cells using propidium iodide (PI) and trypan blue (TB) staining followed by flow cytometry analysis after 5 days of treatment. As shown in Figure 1A, Dac significantly increased cell death with dose.

Next, we tested whether *MYCN*-amplification affects NB cells susceptibility to Dac. *MYCN*-non-amplified SK-N-AS and *MYCN*-amplified SK-N-DZ human NB cells were treated with 2.5-μM Dac for 5 days. As shown in Figure 1B, Dac significantly increased the death rate in SK-N-AS cells up to 8-fold and tripled the death rate in SK-N-DZ cells (*p* < 0.001 and *p* < 0.01, respectively). SK-N-AS cells were more sensitive to Dac treatment (*p* < 0.001, Figure 1B). Double-staining with annexin V/PI indicated that Dac treatment induces both early and late apoptosis significantly. (Supplementary Figure S1A). To clarify the underlying mechanism, we utilized microarray to analyze the differential gene expression in SK-N-AS cells in response to Dac (Supplementary Figure S1B). As shown in Figure 1C, treatment with Dac induced some interferon-stimulated genes (ISGs), including *DDX58,* which encodes RIG-I, a dsRNA sensor for initiating innate immune response.

Then we evaluated whether Dac could modify *DDX58*/RIG-I at the epigenetic level. After treatment with Dac, the expression of DNA methyltransferase 1 (DNMT1) protein was decreased with different dose (Figure 1D). We evaluated the methylation level of *DDX58* promoter in four selected CpG sites (Supplementary Figure S2) using bisulfite pyrosequencing. As shown in Figure 1E, Dac suppressed the methylation level of *DDX58* promoter. The results suggest that Dac affects the expression of DNMT1, leading to the decreased methylation of *DDX58*/RIG-I.

Dac has been reported to stimulate the expression of endogenous dsRNA [16,17], so we explored the role of Dac in inducing dsRNA in NB cells. The monoclonal antibody J2 (for dsRNA detection) and anti-Tom20 (mitochondria inner membrane) were used to examine spatial distribution and the production of dsRNA. In the untreated control (NT) group, the dsRNA signal expressed in a faint intensity (Figure 1F) and could be observed within mitochondria (Supplementary Figure S3A). In contrast, Dac-treated cells showed a stronger dsRNA expression, particularly in the cytosol (Figure 1F). The quantification of dsRNA fluorescence signal was investigated under lower magnification from three independent experiments (Figure 1F’ and Supplementary Figure S3B). These results suggest that Dac may induce endogenous dsRNA and activate RIG-I, resulting in innate immune-related response.

Four different NB cells, including two *MYCN* non-amplified cells (SK-N-AS and SK-N-FI) and two *MYCN* amplified cells (BE(2)M17 and SK-N-DZ) (Figure 1G) were used to clarify the innate immunity response of Dac. Dac treatment induced marked RIG-1 protein expression in SK-N-AS and SK-N-FI cells. Furthermore, such RIG-1 related downstream proteins as MAVS and phosphorylated IRF-3 (p-IRF3) were also detected only in SK-N-AS cells (Figure 1G). Moreover, the apoptosis indicator cleaved caspase-9 in SK-N-AS cells was detected after the treatment of Dac (Figure 1G). However, both no-treated and Dac-treated SK-N-FI cells presented a similar level of cleaved caspase-9 with seldom phosphorylation of IRF-3 (Figure 1G). In addition, the apoptotic rate of SK-N-FI cells remained unchanged after Dac treatment (Supplementary Figure S3C). These findings indicate Dac-induced cell apoptosis involved in the activation of the RIG-1 pathway in SK-N-AS cells only. In line with our previous study, SK-N-AS, but not SK-N-FI and SK-N-DZ cells, exhibited activated innate immunity signaling and marked apoptosis in response to immunostimulant stimulation [23]. Thereafter, we focused on the implication of Dac-induced innate immunity signaling in triggering apoptosis of SK-N-AS cells.

**Figure 2:**
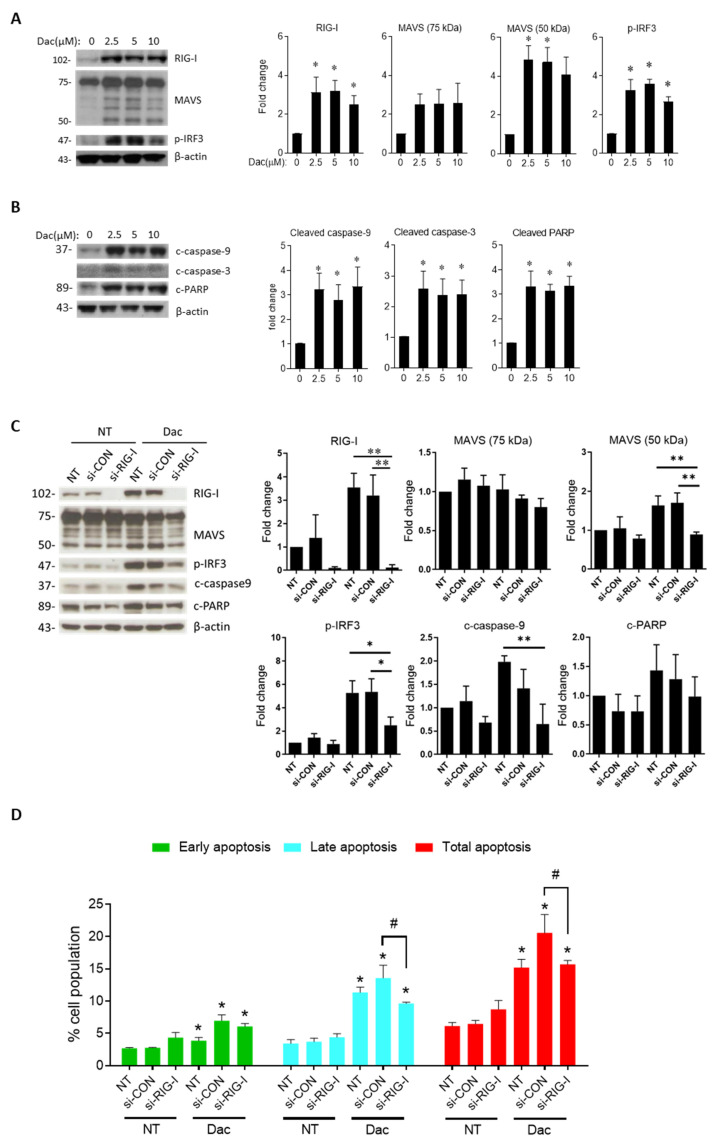
Dose manner of innate immune response and apoptosis signaling under Dac treatment. For dose manner test, SK-N-AS cells were treated with Dac from 0 to 10 μM for 5 days. For siRNA-treated experiments, SK-N-AS cells were treated with or without 2.5-μM Dac for 5 days. Representative western blot result and histogram of densitometric quantification data of innate immune response markers RIG-I, MAVS and p-IRF3 (**A**) and apoptotic signaling cleaved caspase-9, -3 and poly [ADP-ribose] polymerase (PARP) (**B**); β-actin serves as loading control; (**C**) cells transfected with siRNA control sequence (si-con) or siRNA-targeting RIG-I (si-RIG-I) or no treatment (NT) for 1 day followed by being treated with 2.5-μM Dac for 5 days. Representative western blot results of RIG-I, MAVS, p-IRF3, cleaved caspase-9 and PARP are shown. β-actin serves as the loading control. * *p* < 0.05, ** *p* < 0.01 between indicated groups; (**D**) Cell apoptosis rate was measured using annexin V/PI staining by flow cytometry from at least three independent experiments. * *p* < 0.05 Dac group compared with corresponding NT group. ^#^ *p* < 0.05 between indicated groups. NT—untreated.

Mitochondrial antiviral signaling protein ubiquitination and degradation is a vital step for activating downstream innate immune response [24]. In our experiment, smaller degraded MAVS isoform (50 kDa) was detected in AS and FI cells under Dac stimulation, while the expression of undegraded MAVS protein was found in BE (2)M17 and SK-N-DZ cells (Figure 1G). We verified the degraded pattern of MAVS in the context of provoked innate immune response in SK-N-AS cells by the use of poly (I:C) (Supplementary Figure S4). A dose manner test revealed that Dac at 2.5-μM is an effective dose for triggering innate immune signaling and apoptotic response in SK-N-AS cells (Figure 2A,B).

Then, we verified the role of *DDX58*/RIG-I by siRNA knockdown and found that inhibition of RIG-I significantly attenuated the degraded form of MAVS, p-IRF3 and cleaved caspase-9 (Figure 2C). Attenuated RIG-1 gene expression also suppressed the Dac-induced late phase cell apoptosis in SK-N-AS cells (Figure 2D). As such, these results indicate that RIG-I acts as one of the positive regulators in mediating the effect of Dac on innate immune signaling and NB apoptosis.

### 3.2. mtDNA Plays a Vital Role in Dac-Activated Innate Immune Response and Apoptosis

**Figure 3:**
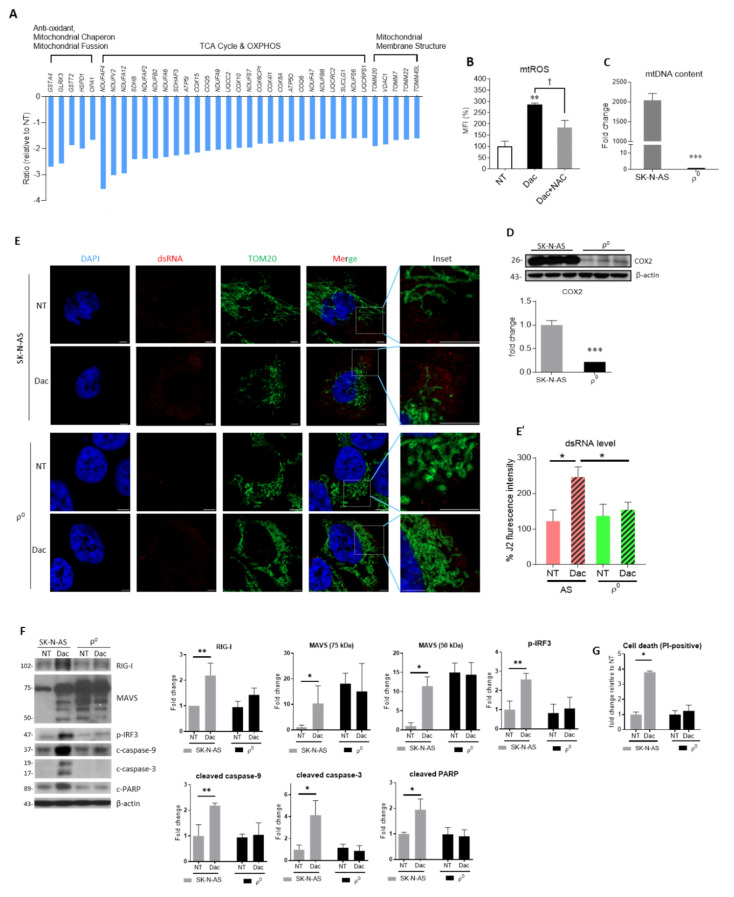
mtDNA is required for Dac-activated dsRNA abundance and innate immune response and apoptosis. (**A**) SK-N-AS cells treated with 2.5 μM Dac or NT for 5 days were harvested and subjected to microarray analysis. Histogram showing down-regulated mitochondrial genes regarding anti-oxidant, chaperon, fusion, tricarboxylic acid cycle (TCA) cycle, oxidative phosphorylation (OXPHOS) and mitochondrial membrane structure; (**B**) mitochondrial ROS (mtROS) probed by MitoSox^TM^ red staining and detected by a flow cytometry was expressed as percentage of mean fluorescence intensity (MFI). Co-treatment of 10-mM N-acetylcysteine (NAC) for 5 days serves as a ROS scavenger control; (**C**) mtDNA content of SK-N-AS and ρ^0^ cells was measured using qRT-PCR; (**D**) representative western blot results and densitometric quantification data of mtDNA-encoded COX2. β-actin serves as the loading control for normalization. *** *p* < 0.001 compared to SK-N-AS cells. (**E**) representative immunofluorescent image of dsRNA (red), mitochondrial outer membrane marker TOM20 (green) and DAPI-stained nuclei (blue) in ρ^0^ cells. Scale bar, 5 μm. Insets contain magnified images highlighting mitochondria and dsRNA. (**E’**) percentage of J2 fluorescence intensity (a.u., arbitrary unit) was quantified (representative images shown in Supplementary Figure S6B) under lower magnification (100X) of an Olympus FV10i confocal microscope. * *p* < 0.05 between indicated groups; (**F**) representative western blot of RIG-I, MAVS, p-IRF3, cleaved caspase-9, -3 and PARP are shown. β-actin serves as the loading control; (**G**) cell death rate was detected using PI or TB staining with flow cytometry. * *p* < 0.05, ** *p* < 0.01 when compared to NT or parental cell group. ^†^ *p* < 0.05 between indicated groups. NT—untreated; MFI—mean fluorescence intensity.

In our microarray data, we also found a series of downregulated mitochondrial genes following Dac treatment, including genes related to anti-oxidant, chaperone and mitochondrial dynamics (Figure 3A), which are critical to oxidative stress [25]. As oxidative stress is characterized by overproduction of reactive oxygen species (ROS) that can cause damage to mitochondrial structure and function [26], we evaluated mitochondrial ROS (mtROS) by using MitoSox^TM^ Red. As shown in Figure 3B, Dac induced a significant increase in mtROS. This induced mtROS by Dac was significantly suppressed in the presence of ROS scavenger N-acetylcysteine (NAC) (Figure 3B). Attenuation of mitochondrial membrane potential by the treatment of Dac indicates impaired mitochondrial integrity (Supplementary Figure S5A). As mitochondrial import machinery is implicated in regulating the state of mitochondrial oxidative stress in NB cells [27], we further assess the TOM20, a mitochondrial translocase of outer membrane. To validate the results from microarray and mt-ROS measurement (Figure 3A,B), we checked the protein expression of TOM20 level and confirmed the expression of TOM20 was significantly reduced in response to Dac (Supplementary Figure S5B).

Since mtDNA can trigger the innate immune response, we sought to clarify its role by generating mtDNA-depleted SK-N-AS cells (AS-ρ^0^ cells). After long term exposure of ethidium bromide along with supplementation of pyruvate and uridine, AS ρ^0^ cells presented devoid of mtDNA (Figure 3C), as well as greatly reduced mtDNA-encoded protein cytochrome c oxidase 2 (COX2) (Figure 3D). To confirm whether exposure of ethidium bromide leads to DNA damage of nuclear genome which may trigger transformation of cellular nature, we compared the level of phosphorylation of γH2AX at Serine 139 in SK-N-AS and AS-ρ^0^ cells. As shown in Supplementary Figure S6A, SK-N-AS and AS-ρ^0^ cells manifested similar level of γH2AX (p-S139). Unlike strikingly increased level of endogenous dsRNA in Dac-treated SK-N-AS cells, AS-ρ^0^ cells presented an unchanged dsRNA level following Dac stimulation (Figure 3E,E’; Supplementary Figure S6B).

AS-ρ^0^ cells treated with Dac also demonstrated an attenuated response in RIG-I, MAVS, p-IRF3, cleaved caspase-9, -3 and PARP (Figure 3F). The cell death of AS-ρ^0^ cells was not affected by Dac, compared to the original SK-N-AS cells (Figure 3G). These results verify that functional mitochondria are required to convey Dac-induced innate immune response and apoptotic cell death in SK-N-AS NB cells. However, the exact mechanism of mtDNA involving the Dac-induced effect in NB cells needs further investigation.

### 3.3. Dac Potentiates Anti-NB Effect of CDDP and Poly(I:C)

**Figure 4:**
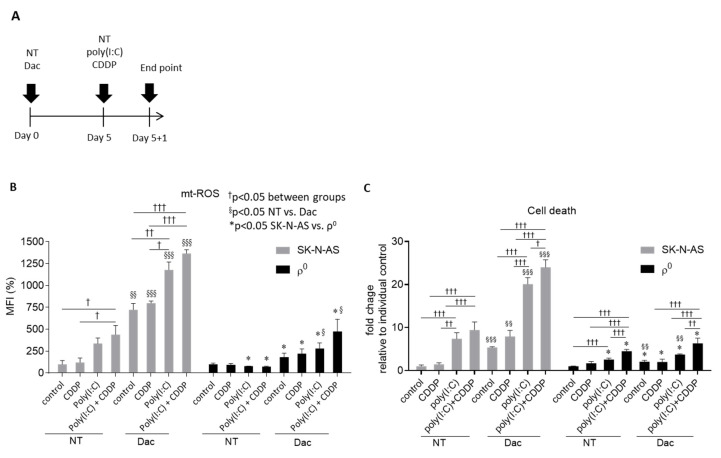
Dac potentiates mtROS-provoking and anti-NB effect of CDDP and poly(I:C). (**A**) Experimental flow chart of drug combinations; (**B**) mitochondrial ROS (mtROS) probed by MitoSox^TM^ Red staining and detected by flow cytometry; (**C**) cell death rate was detected using PI with flow cytometry. * *p* < 0.05 between SK-N-AS and ρ^0^ cells. ^†^ *p* < 0.05, ^††^ *p* < 0.01, ^†††^ *p* < 0.001 between indicated groups. ^§^ *p* <0.05, ^§§^ *p* <0.01, ^§§§^ *p* < 0.001 between NT and Dac. NT—untreated; MFI—mean fluorescence intensity.

Cisplatin (CDDP) is a chemotherapeutic agent commonly used to treat NB [28]. Poly(I:C) is also a well-known immunostimulant, enabling activation of innate immune response through TLR3 [21]. We examined whether Dac can augment the effects of cisplatin and/or poly(I:C) on NB. Cells were pretreated with 2.5-μM Dac for 5 days, followed by exposure to 10-μM CDDP, 50-μg/mL poly(I:C) or both for 24 h (Figure 4A). Treatment with poly I:C alone or combination of poly I:C and CDDP could induce higher ROS production (Figure 4B) and death rate in AS cells (Figure 4C). However, additional Dac stimulation strikingly augmented mitochondrial ROS level in all groups (Figure 4B), while AS ρ^0^ cells devoid of mtDNA greatly attenuated this ROS production with a limited cell death rate (Figure 4C). The therapeutic effect of Dac with combination with other agents induced more cell death through increasing ROS production.

## 4. Discussion

In this study, we demonstrated that Dac treatment induces cell death on *MYCN*-non-amplified NB cells through activation of the RIG-I-related innate immune response, which involves decreasing methylated *DDX58* promoter and the release of endogenous dsRNA. By modulating mitochondrial ROS production, Dac enhanced the cytotoxic effect of poly I:C and CDDP on NB cells.

As a DNMT inhibitor, Dac presents broad effects in inducing cell differentiation and reduced proliferation in NB [29]. Although Dac has been shown to provoke innate immune signaling to exert an antitumoral effect [16,17], whether *MYCN* amplification hampers such effects of Dac on NB cells remains unknown. In fact, *MYCN* amplification has been shown to repress cellular immunity, while *MYCN* deletion was shown to restore the innate immune response [30,31]. The results of our previous study suggest that NB with *MYCN* amplification shows resistance to immunostimulant treatment. In this study, *MYCN* non-amplified NB cells showed more susceptible to Dac treatment than those with *MYCN* amplification, indicating that the presence of *MYCN* serves as a resistance factor for treatment response.

Cytosolic PRRs such as RIG-I, MDA5 or TLR3 may potentially account for Dac-mediated immune response. Activation of PRRs—along with MAVS—together trigger the nuclear translocation of IRF3/7 to turn on interferon-responsive genes. Under Dac treatment, these PRRs are activated and implicated in the cell death of human bronchial epithelial cells [32], ovarian cancer cells [17] and colon cancer cells [16]. In our study, RIG-I, but not MDA5 and TLR3, was significantly increased by Dac treatment, suggesting that RIG-I is the predominant PRR in sensing Dac in *MYCN* non-amplification NB cells. Regarding the antitumor effect of RIG-I, Liu et al. have demonstrated that downregulation of RIG-I in hepatocellular carcinoma is correlated with poor clinical outcome, while in vitro overexpression of RIG-I can enhance the interferon response to suppress proliferation of hepatocellular carcinoma [33]. Interestingly, our results demonstrated that siRNA-targeting RIG-I reverses Dac-induced innate immune response and cell apoptosis, highlighting the implication of RIG-I in epigenetic modulation of NB treatment.

In our study, we found that the DNMT1-inhibiting activity of Dac causes the hypomethylated status of *DDX58*/RIG-I promoter, leading to its overexpression. The involvement of mtROS in innate immune responses has been reviewed in much literature [34]. Agod et al. have reported that mtROS play a central role in stimulating RIG-I-mediated interferon response in immune cells [35]. Herein, we found that Dac increases mtROS in NB via an imbalanced expression profile of mitochondrial genes. On the other hand, endogenous dsRNA could play a role in activating RIG-I and its downstream immune signaling. Roulois et al. described that Dac treatment in colon cancer leads to an increase in transcription of endogenous retrovirus, which generates intracellular dsRNA [16]. Similar findings were exhibited by Chiappinelli et al. [17]. Meanwhile, Dhir et al. brought up another insight that more than 95% of endogenous dsRNA comes from mitochondria, as evidenced by J2 antibody-based immunoprecipitation along with RNA seq. These cytosolic dsRNAs act to trigger innate immune signaling dependent on MDA5 and partly on RIG-I. In our study, we observed dsRNA colocalized with mitochondria in untreated cells, while cytoplasmic dsRNA level was shown to increase in Dac-treated NB cells. Notably, we did not observe this phenomenon in mtDNA-depleted ρ^0^ cells, suggesting the possibility that Dac-induced dsRNA may be of mitochondrial origin. The lack of exact identification of these dsRNA limits out study in explaining their origin.

The use of ρ^0^ cells enables to clarifying that mtDNA is important in Dac-induced RIG-I, dsRNA accumulation as well as cell death. However, these cells are metabolically different from their counterparts and inefficient mitochondria such as ρ^0^ cells have been largely involved in cancer resistance [36,37,38,39]. Gonzalez-Sanchez et al. have reported that ρ^0^ cells of hepatocellular carcinoma exhibit a reduction in Bax/Bcl-2 ratio in the presence of chemotherapeutic drugs [36]. It suggested that altered metabolic phenotype may result in the modification of mitochondria-mediated apoptotic signals to acquire drug tolerance ultimately. Similarly, in this study, ρ^0^ cells of SK-N-AS NB developed more resistance against Dac and treatment combinations with CDDP and/or poly(I:C). Nevertheless, further study to decipher the detail molecular underpinning the resistance of ρ^0^ cells is warranted, and its clarification will provide further insights into the NB treatment strategy.

Mitochondria have been shown to be a preferential target of the chemotherapeutic drug CDDP and immunostimulant poly(I:C). CDDP can cause cancer cell apoptosis by binding to mtDNA and the voltage-dependent anion channel 1 (VDAC1) of the mitochondrial outer membrane [40,41]. Our previous study demonstrated that mtDNA is required for mtROS production induced by poly(I:C) [21]. In the present study, we discovered that Dac increased mtROS by repressing the expression of mitochondrial genes that preserve redox homeostasis and further potentiates mtROS production and the anti-NB effect of CDDP and poly(I:C). Of particular note, a lack of mtDNA greatly diminished this effect, indicating the crucial role of functional mitochondria in the Dac treatment of NB cells. Therefore, we suggest that disturbing mitochondrial oxidative stress could be a future therapeutic strategy for the clinical application of Dac in patients with NB.

## 5. Conclusions

This study demonstrates that Dac effectively induces a RIG-I-related innate immune response and apoptotic signaling in *MYCN* non-amplified NB cells through the hypomethylation of the *DDX58*/RIG-I promoter and elevated mtROS with increased dsRNA. Furthermore, Dac can potentiate the cytotoxic effects of CDDP and poly(I:C) on NB cells.

## Supplementary Materials

The following are available online at https://www.mdpi.com/2073-4409/9/9/1920/s1, Figure S1. (related to Figure 1B–C); Figure S2. Pyrosequencing design for four sites of the *DDX58*/RIG-I promoter; Figure S3; Figure S4. MAVS expression pattern in response to Poly(I:C); Figure S5. Loss of mitochondrial membrane potential and reduction of TOM20 protein level in response to Dac; Figure S6. AS and ρ^0^ cells manifest a similar level of DNA damage marker γH2AX (p-S139), but differ in the intracellular dsRNA level; Table S1. Primer sequences used for pyrosequencing.

## References

[1] Maris J.M., Hogarty M.D., Bagatell R., Cohn S.L. (2007). Neuroblastoma. Lancet.

[2] Maris J.M. (2010). Recent advances in neuroblastoma. N. Engl. J. Med..

[3] Liu X., Mazanek P., Dam V., Wang Q., Zhao H., Guo R., Jagannathan J., Cnaan A., Maris J.M., Hogarty M.D. (2008). Deregulated Wnt/beta-catenin program in high-risk neuroblastomas without MYCN amplification. Oncogene.

[4] Ng J.M., Yu J. (2015). Promoter hypermethylation of tumour suppressor genes as potential biomarkers in colorectal cancer. Int. J. Mol. Sci..

[5] Philipp A.B., Stieber P., Nagel D., Neumann J., Spelsberg F., Jung A., Lamerz R., Herbst A., Kolligs F.T. (2012). Prognostic role of methylated free circulating DNA in colorectal cancer. Int. J. Cancer.

[6] Herbst A., Wallner M., Rahmig K., Stieber P., Crispin A., Lamerz R., Kolligs F.T. (2009). Methylation of helicase-like transcription factor in serum of patients with colorectal cancer is an independent predictor of disease recurrence. Eur. J. Gastroenterol. Hepatol..

[7] Wallner M., Herbst A., Behrens A., Crispin A., Stieber P., Goke B., Lamerz R., Kolligs F.T. (2006). Methylation of serum DNA is an independent prognostic marker in colorectal cancer. Clin. Cancer Res..

[8] Gomez S., Castellano G., Mayol G., Sunol M., Queiros A., Bibikova M., Nazor K.L., Loring J.F., Lemos I., Rodriguez E. (2015). DNA methylation fingerprint of neuroblastoma reveals new biological and clinical insights. Epigenomics.

[9] Abe M., Ohira M., Kaneda A., Yagi Y., Yamamoto S., Kitano Y., Takato T., Nakagawara A., Ushijima T. (2005). CpG island methylator phenotype is a strong determinant of poor prognosis in neuroblastomas. Cancer Res..

[10] Yang Q., Tian Y., Ostler K.R., Chlenski A., Guerrero L.J., Salwen H.R., Godley L.A., Cohn S.L. (2010). Epigenetic alterations differ in phenotypically distinct human neuroblastoma cell lines. BMC Cancer.

[11] Tsai H.C., Li H., Van Neste L., Cai Y., Robert C., Rassool F.V., Shin J.J., Harbom K.M., Beaty R., Pappou E. (2012). Transient low doses of DNA-demethylating agents exert durable antitumor effects on hematological and epithelial tumor cells. Cancer Cell.

[12] Carpinelli P., Granata F., Augusti-Tocco G., Rossi M., Bartolucci S. (1993). Antiproliferative effects and DNA hypomethylation by 5-aza-2′-deoxycytidine in human neuroblastoma cell lines. Anticancer Drugs.

[13] Bartolucci S., Estenoz M., Longo A., Santoro B., Momparler R.L., Rossi M., Augusti-Tocco G. (1989). 5-Aza-2′-deoxycytidine as inducer of differentiation and growth inhibition in mouse neuroblastoma cells. Cell Differ. Dev..

[14] Charlet J., Schnekenburger M., Brown K.W., Diederich M. (2012). DNA demethylation increases sensitivity of neuroblastoma cells to chemotherapeutic drugs. Biochem. Pharmacol..

[15] Navada S.C., Steinmann J., Lubbert M., Silverman L.R. (2014). Clinical development of demethylating agents in hematology. J. Clin. Investig..

[16] Roulois D., Loo Yau H., Singhania R., Wang Y., Danesh A., Shen S.Y., Han H., Liang G., Jones P.A., Pugh T.J. (2015). DNA-Demethylating Agents Target Colorectal Cancer Cells by Inducing Viral Mimicry by Endogenous Transcripts. Cell.

[17] Chiappinelli K.B., Strissel P.L., Desrichard A., Li H., Henke C., Akman B., Hein A., Rote N.S., Cope L.M., Snyder A. (2015). Inhibiting DNA Methylation Causes an Interferon Response in Cancer via dsRNA Including Endogenous Retroviruses. Cell.

[18] Banoth B., Cassel S.L. (2018). Mitochondria in innate immune signaling. Transl. Res..

[19] Wiatrek D.M., Candela M.E., Sedmik J., Oppelt J., Keegan L.P., O’Connell M.A. (2019). Activation of innate immunity by mitochondrial dsRNA in mouse cells lacking p53 protein. RNA.

[20] Dhir A., Dhir S., Borowski L.S., Jimenez L., Teitell M., Rotig A., Crow Y.J., Rice G.I., Duffy D., Tamby C. (2018). Mitochondrial double-stranded RNA triggers antiviral signalling in humans. Nature.

[21] Chuang J.H., Lin T.K., Tai M.H., Liou C.W., Huang S.T., Wu C.L., Lin H.Y., Wang P.W. (2012). Preferential involvement of mitochondria in Toll-like receptor 3 agonist-induced neuroblastoma cell apoptosis, but not in inhibition of cell growth. Apoptosis.

[22] Ikegaki N., Shimada H., Fox A.M., Regan P.L., Jacobs J.R., Hicks S.L., Rappaport E.F., Tang X.X. (2013). Transient treatment with epigenetic modifiers yields stable neuroblastoma stem cells resembling aggressive large-cell neuroblastomas. Proc. Natl. Acad. Sci. USA.

[23] Chuang J.H., Chuang H.C., Huang C.C., Wu C.L., Du Y.Y., Kung M.L., Chen C.H., Chen S.C., Tai M.H. (2011). Differential toll-like receptor 3 (TLR3) expression and apoptotic response to TLR3 agonist in human neuroblastoma cells. J. Biomed. Sci..

[24] Castanier C., Zemirli N., Portier A., Garcin D., Bidere N., Vazquez A., Arnoult D. (2012). MAVS ubiquitination by the E3 ligase TRIM25 and degradation by the proteasome is involved in type I interferon production after activation of the antiviral RIG-I-like receptors. BMC Biol..

[25] Moehle E.A., Shen K., Dillin A. (2019). Mitochondrial proteostasis in the context of cellular and organismal health and aging. J. Biol. Chem..

[26] Murphy M.P. (2009). How mitochondria produce reactive oxygen species. Biochem. J..

[27] Franco-Iborra S., Cuadros T., Parent A., Romero-Gimenez J., Vila M., Perier C. (2018). Defective mitochondrial protein import contributes to complex I-induced mitochondrial dysfunction and neurodegeneration in Parkinson's disease. Cell Death Dis..

[28] Bomken S., Davies B., Chong L., Cole M., Wood K.M., McDermott M., Tweddle D.A. (2011). Percentage tumor necrosis following chemotherapy in neuroblastoma correlates with MYCN status but not survival. Pediatr. Hematol. Oncol..

[29] Jubierre L., Jimenez C., Rovira E., Soriano A., Sabado C., Gros L., Llort A., Hladun R., Roma J., Toledo J.S. (2018). Targeting of epigenetic regulators in neuroblastoma. Exp. Mol. Med..

[30] Zhang P., Wu X., Basu M., Dong C., Zheng P., Liu Y., Sandler A.D. (2017). MYCN Amplification Is Associated with Repressed Cellular Immunity in Neuroblastoma: An In Silico Immunological Analysis of TARGET Database. Front. Immunol..

[31] Layer J.P., Kronmuller M.T., Quast T., van den Boorn-Konijnenberg D., Effern M., Hinze D., Althoff K., Schramm A., Westermann F., Peifer M. (2017). Amplification of N-Myc is associated with a T-cell-poor microenvironment in metastatic neuroblastoma restraining interferon pathway activity and chemokine expression. Oncoimmunology.

[32] Wu W., Zhang W., Booth J.L., Hutchings D.C., Wang X., White V.L., Youness H., Cross C.D., Zou M.H., Burian D. (2016). Human primary airway epithelial cells isolated from active smokers have epigenetically impaired antiviral responses. Respir Res..

[33] Liu Z., Dou C., Jia Y., Li Q., Zheng X., Yao Y., Liu Q., Song T. (2015). RIG-I suppresses the migration and invasion of hepatocellular carcinoma cells by regulating MMP9. Int. J. Oncol..

[34] Pourcelot M., Arnoult D. (2014). Mitochondrial dynamics and the innate antiviral immune response. FEBS J..

[35] Agod Z., Fekete T., Budai M.M., Varga A., Szabo A., Moon H., Boldogh I., Biro T., Lanyi A., Bacsi A. (2017). Regulation of type I interferon responses by mitochondria-derived reactive oxygen species in plasmacytoid dendritic cells. Redox Biol..

[36] Gonzalez-Sanchez E., Marin J.J., Perez M.J. (2014). The expression of genes involved in hepatocellular carcinoma chemoresistance is affected by mitochondrial genome depletion. Mol. Pharm..

[37] Montopoli M., Bellanda M., Lonardoni F., Ragazzi E., Dorigo P., Froldi G., Mammi S., Caparrotta L. (2011). “Metabolic reprogramming” in ovarian cancer cells resistant to cisplatin. Curr. Cancer Drug Targets.

[38] Qian W., Nishikawa M., Haque A.M., Hirose M., Mashimo M., Sato E., Inoue M. (2005). Mitochondrial density determines the cellular sensitivity to cisplatin-induced cell death. Am. J. Physiol. Cell Physiol..

[39] Park S.Y., Choi B., Cheon H., Pak Y.K., Kulawiec M., Singh K.K., Lee M.S. (2004). Cellular aging of mitochondrial DNA-depleted cells. Biochem. Biophys. Res. Commun..

[40] Tajeddine N., Galluzzi L., Kepp O., Hangen E., Morselli E., Senovilla L., Araujo N., Pinna G., Larochette N., Zamzami N. (2008). Hierarchical involvement of Bak, VDAC1 and Bax in cisplatin-induced cell death. Oncogene.

[41] Yang Z., Schumaker L.M., Egorin M.J., Zuhowski E.G., Guo Z., Cullen K.J. (2006). Cisplatin preferentially binds mitochondrial DNA and voltage-dependent anion channel protein in the mitochondrial membrane of head and neck squamous cell carcinoma: Possible role in apoptosis. Clin. Cancer Res..

