# Supplementary material for: 5-aza-2′-Deoxycytidine Induces a RIG-I-Related Innate Immune Response by Modulating Mitochondria Stress in Neuroblastoma
Source: Cells. 2020 Aug 19;9(9):1920. doi: 10.3390/cells9091920 (PMC7564572; doi:10.3390/cells9091920)
Supplement: Supplementary file 1 [file cells-09-01920-s001.pdf]

Supplementary materials

A

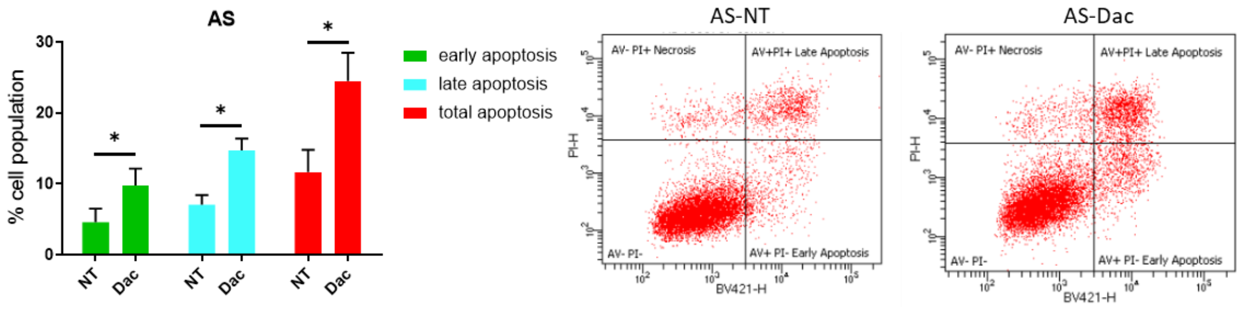

B

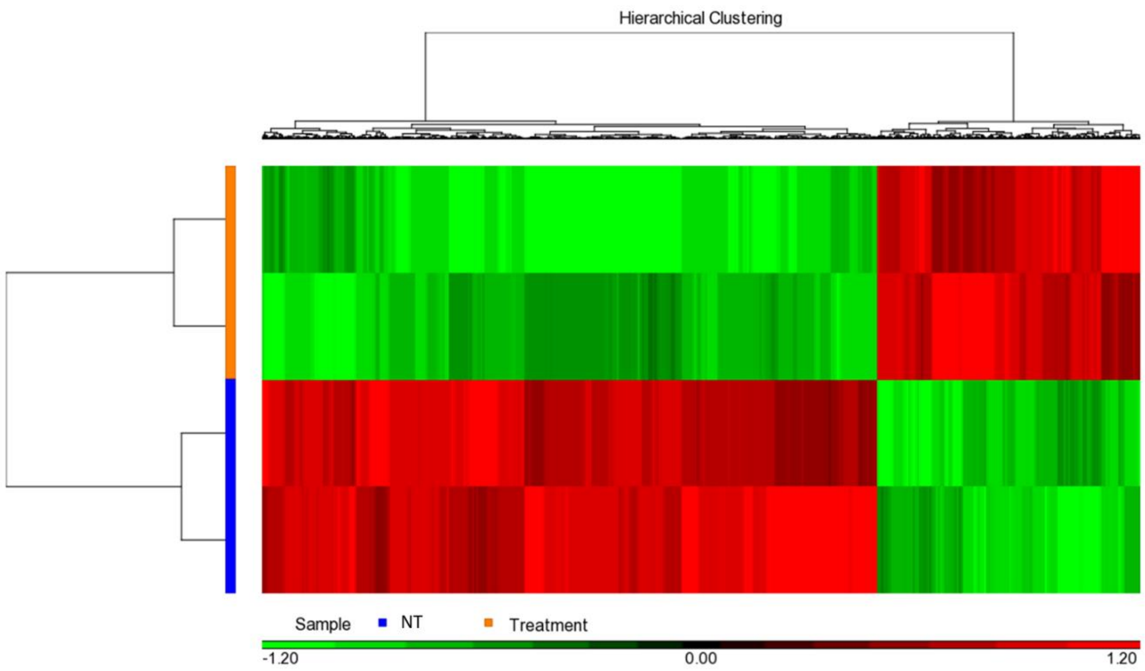

Figure S1. (related to Figure 1B-C): (A) SK-N-AS cell apoptosis was determined by double staining with annexinV/PI by flow cytometry. *p<0.05 compared with the indicated group. (B) Hierarchical clustering of SK-N-AS cells based on expression values of genes selected by standardize analysis. The analysis showed the expression of genes in no-treated (NT) SK-N-AS cells (orange) and Dac-treated SK-N-AS cells (blue, 2.5 μM for 5 d). No treated control (NT) and Dac-treated groups were pooled (n=3 and 6 for control and Dac, respectively).

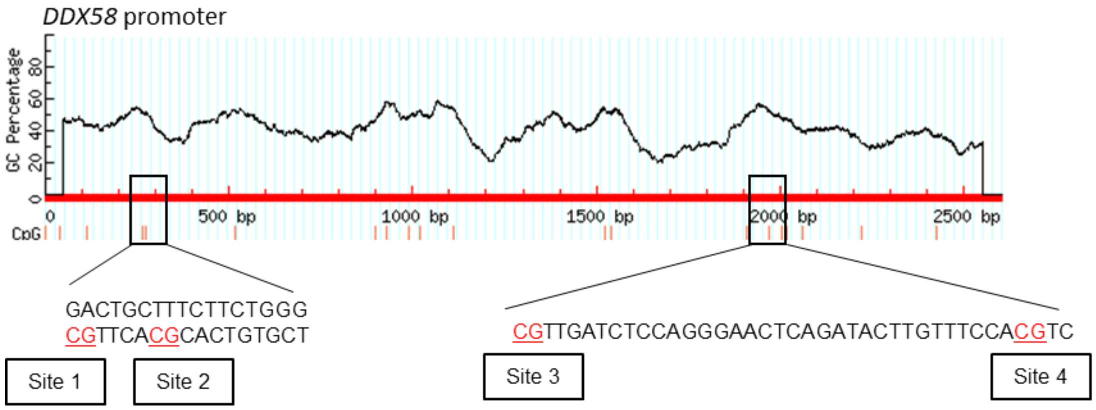

Figure S2. (related to Figure 1) Pyrosequencing design for four sites of the *DDX58*/RIG-I promoter. Red underlines denote CpG sites.

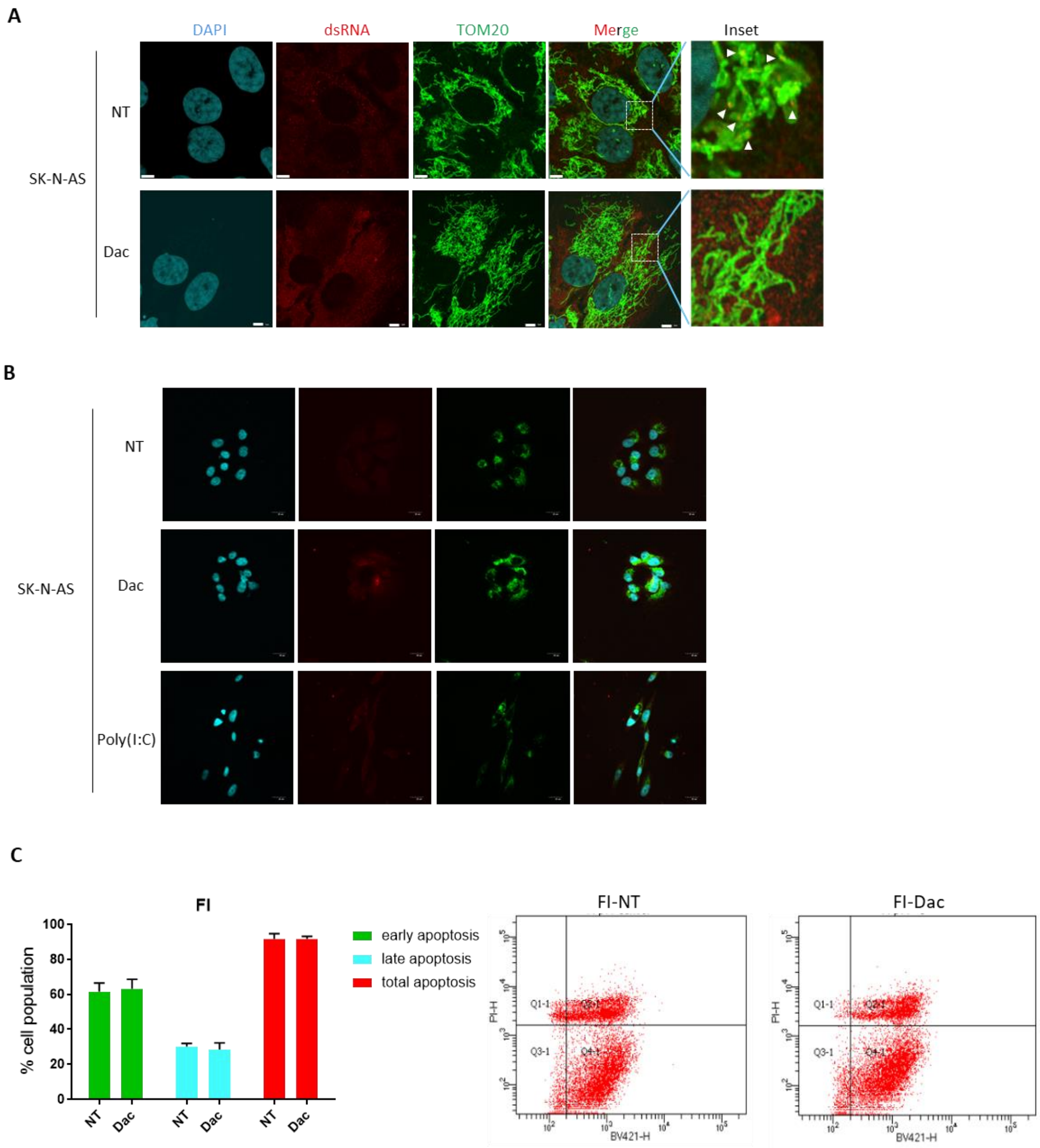

Figure S3. (A) Related to Figure 1F. Representative immunofluorescent image of dsRNA (red), mitochondrial outer membrane marker TOM20 (green), and DAPI-stained nuclei (blue). Scale bar, 5 µm. Insets contain magnified images highlighting mitochondria and dsRNA. Arrowheads indicate dsRNA within mitochondria. Note that Dac treatment causes an increased level of dsRNA in the cytoplasm. (B) Related to Figure 1 F′. The immunofluorescent statin of the dsRNA signal was investigated under lower magnification (100X) of the Olympus FV10i confocal microscope. dsRNA, mitochondria, and nucleus probed by J2 antibody (red), anti-TOM20 (green), and DAPI (blue), respectively. The result of the quantification of dsRNA signal intensity was shown in Figure 1F′. (C) Related to Figure 1G. The cell apoptosis rate of SK-N-FI cells from at least three independent experiments was stained using annexin V/PI by flow cytometry. Dac treatment didn’t affect the cell apoptosis in SK-N-FI cells.

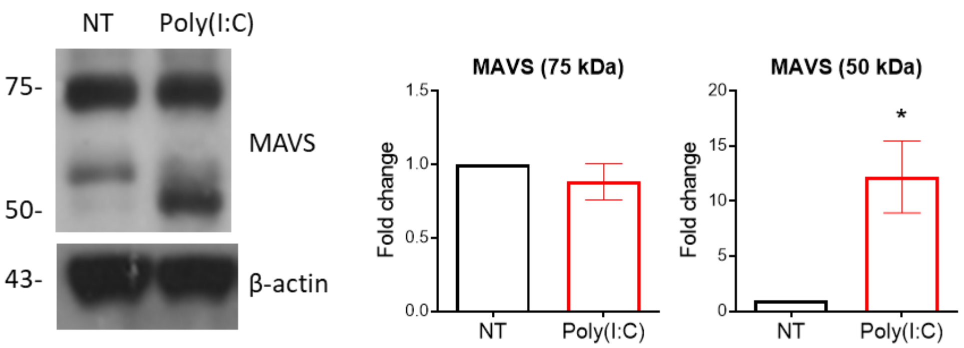

Figure S4. MAVS expression pattern in response to Poly(I:C). Representative immunoblot image of the larger and smaller degraded form of MAVS (75 and 50 kDa, respectively). SK-N-AS cells were either not treated (NT) or treated with 50 µg/ml Poly(I:C) for 24 h. β-actin was used as the loading control. N=3. *p<0.05 compared with NT.

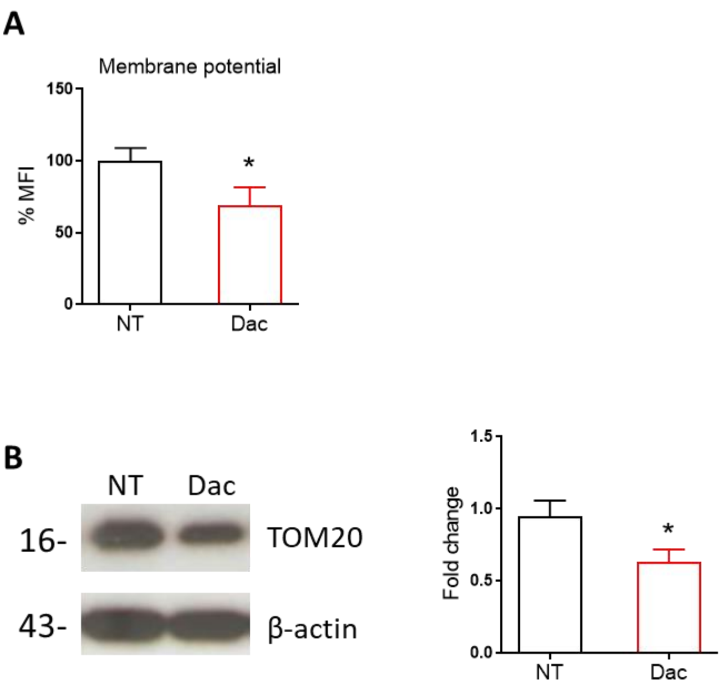

Figure S5. Loss of mitochondrial membrane potential and reduction of TOM20 protein level in response to Dac. (A) Mitochondrial membrane potential was stained by TMRE with flow cytometry. MFI, mean fluorescence intensity. N=3. *p<0.05 compared with no treated (NT) group. (B) Representative immunoblot image of TOM20 of SK-N-AS cells with or without 2.5 µM Dac for 5 days. β-actin was used as the loading control. N=3. *p<0.05 compared with NT.

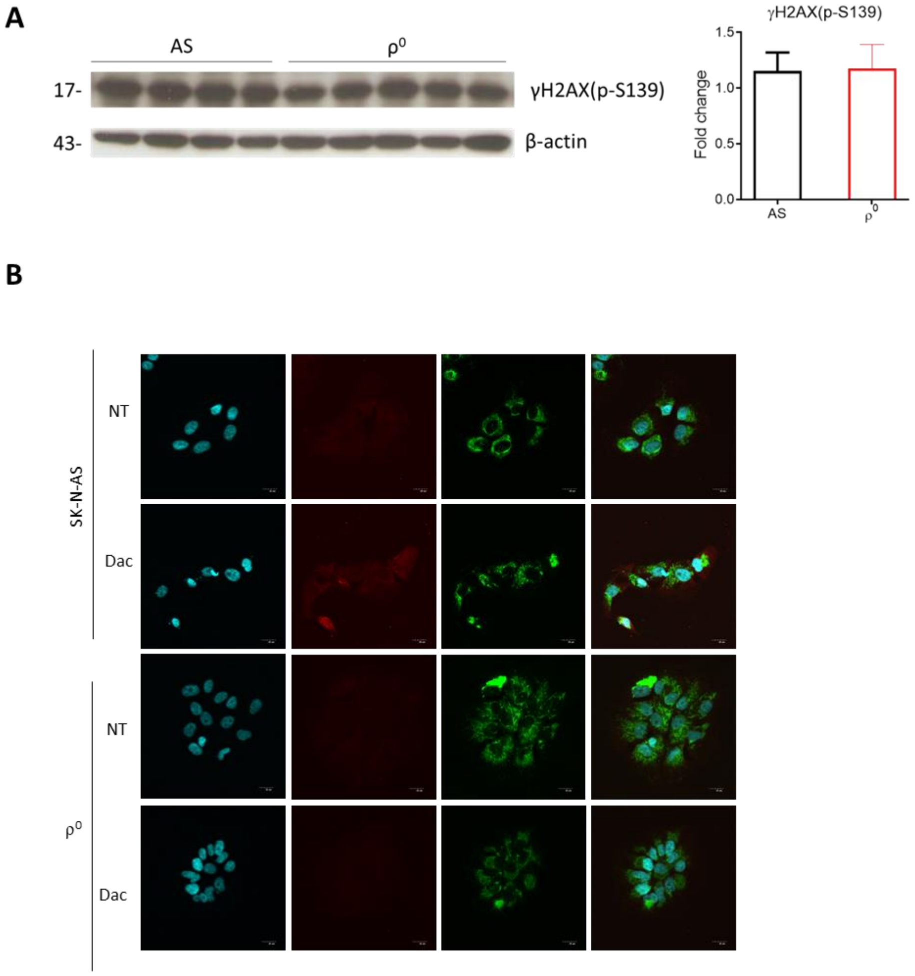

Figure S6. AS and ρ^0 cells manifest a similar level of DNA damage marker γH2AX (p-S139), but differ in the intracellular dsRNA level. (A) Representative immunoblot of γH2AX (p-S139). β-actin was used as the loading control and normalization for densitometric quantification. (B) The immunofluorescent statin of the dsRNA signal was investigated under lower magnification (100X) of the Olympus FV10i confocal microscope. dsRNA, mitochondria, and nucleus probed by J2 antibody (red), anti-TOM20 (green), and DAPI (blue), respectively. Quantification of dsRNA signal obtaining from three independent experiments was shown in Figure 3E'.

Table S1. Primer sequences used for pyrosequencing

Primer (ID)	Sequence (5’→3’)	Amplicon length
Forward primer #1 (F1)	TTGTTTGTAGGGAAGGTGGTAGTAA	210 bp
Reverse primer #1 (R1)	biotin-AATAAAAAATCATCCCAACATCAAAAT	
Sequencing primer #1 (S1)	GTAAATGGTATAGAAATTTATTTG	
Forward primer #2 (F2)	ATGAAGAGGGAGGTAGTTGTA	189 bp
Reverse primer #2 (R2)	biotin-ATAAAACAATCTAACTATCCTTTCTACT	
Sequencing prime #2 (S2)	AGGGAGGTAGTTGTAAT	
